# Through DNA sensors and hidden mitochondrial effects of SARS-CoV-2

**DOI:** 10.1590/1678-9199-JVATITD-2020-0183

**Published:** 2021-08-23

**Authors:** Vitor Pedro Targhetta, Mariana Abrantes Amaral, Niels Olsen Saraiva Camara

**Affiliations:** 1 Department of Immunology, Institute of Biomedical Sciences (ICB), University of São Paulo (USP), São Paulo, SP, Brazil.; 2 Department of Nephrology, Paulista School of Medicine (EPM), Federal University of São Paulo (Unifesp), São Paulo, SP, Brazil.

**Keywords:** SARS-CoV-2, Mitochondria, Innate receptors, Cytokine storm

## Abstract

The COVID-19 pandemic brought attention to studies about viral infections and their impact on the cell machinery. SARS-CoV-2, for example, invades the host cells by ACE2 interaction and possibly hijacks the mitochondria. To better understand the disease and to propose novel treatments, crucial aspects of SARS-CoV-2 enrolment with host mitochondria must be studied. The replicative process of the virus leads to consequences in mitochondrial function, and cell metabolism. The hijacking of mitochondria, on the other hand, can drive the extrusion of mitochondrial DNA (mtDNA) to the cytosol. Extracellular mtDNA evoke robust proinflammatory responses once detected, that may act in different pathways, eliciting important immune responses. However, few receptors are validated and are able to detect and respond to mtDNA. In this review, we propose that the mtDNA and its detection might be important in the immune process generated by SARS-CoV-2 and that this mechanism might be important in the lung pathogenesis seen in clinical symptoms. Therefore, investigating the mtDNA receptors and their signaling pathways might provide important clues for therapeutic interventions.

## Background

At the end of 2019, a new severe acute respiratory syndrome coronavirus (SARS-CoV-2) emerged, and by the middle of 2020 it was already a worldwide pandemic [1,2]. SARS-CoV-2 is a RNA virus and its viral proteins interact with angiotensin-converting enzyme carboxypeptidase 2 (ACE2) and TMPRSS2 proteins of the host to enter the cells and begin the viral replication causing the COVID-19 disease [3]. The virus is capable of causing a severe acute respiratory syndrome lead by an exacerbated immune response triggering a cytokine storm profile [4].

Several recent data have directly linked SARS-CoV-2 infection with the metabolic status and the mitochondrial function of the host cells, and there is commending evidence about an intrinsic relationship between the SARS-CoV-2 viral cycle and the mitochondrial compartment. Risk groups for COVID-19 comprehend people with metabolic diseases like diabetes, obesity and elder people, that have in common decreased mitochondrial function and metabolic alterations [5,6]. Although the main immune considerations established so far regarding mitochondria are mainly related to the maintenance of metabolism in leukocytes [7,8] and the signaling pathways related to the detection of viral RNA (by RIG-I/MAVS for example), other possible immunomodulatory mechanisms, such as the release of mitochondrial damage associated with molecular patterns (DAMPs) into the intracellular or extracellular environment [9,10], were recently discovered. The main DAMP related to this organelle is mitochondrial DNA (mtDNA), the result of the endosymbiont origin that occurred more than 1 billion years ago [11]. Disruption of mtDNA signaling trough diverse pattern recognition receptors (PRRs) is an evolved strategy for a variety of RNA viruses, like dengue and influenza, among others.

**Figure 1.**
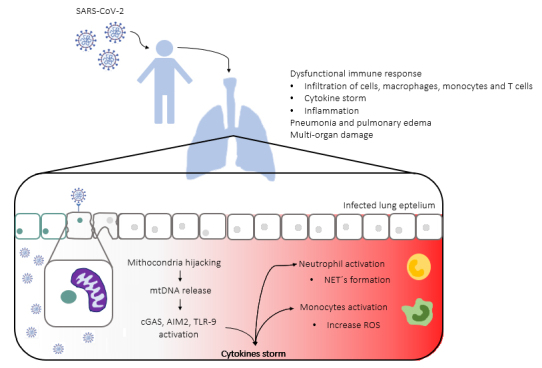
Severe acute respiratory syndrome coronavirus 2 (SARS-CoV-2) lung pathogenesis. SARS-CoV-2 infected cells express the angiotensin-converting enzyme 2 (ACE2) receptor and TMPRSS2, seen in cells of the respiratory tract, among other tissues. The SARS-CoV-2 virus during the replication process hijacks the mitochondria, which leads to the release of mitochondrial DNA (mtDNA) into the cytosol of the cell. The mtDNA presented in the cytosol can be recognized by several receptors as damage-associated molecular patterns (DAMP), such as cGAS, AIM2, TLR9, activating a signal cascade. The signals activated by the DAMP-recognition will be identified by other cells, such as neighbor epithelial cells, endothelial cells, macrophages, among others, triggering a pro-inflammatory response. Meanwhile, the release of mtDNA is also inducing the NETs formation in neutrophils and increasing the production of reactive oxygen species (ROS) in infected monocytes. The activated pro-inflammatory response in different immune cells attracts other cells to the infection site - macrophages, monocytes, T cells - boosting the inflammation and enhancing the cytokine production, which leads to the cytokine storm and ultimately damaging the site. The high concentration of immune cells and the cytokine reach other organs, which eventually causes multi-organ damage.

As discussed throughout this article, mitochondria have different effects on immune responses [12,13] as mtDNA can be directly recognized as a DAMP, and can be linked to systemic inflammation and acute lung injury (Figure 1) [14]. Herein, we highlight a possible novel role for mtDNA in the COVID-19 pathogenesis and give a glimpse about the receptors responsible for its detection and how this can be connected with a worse prognosis. In this review, we hypothesize that mtDNA can affect inflammatory processes and influence SARS-CoV-2 treatment approaches.

## The pathological roles of mtDNA

mtDNA, unlike genomic DNA, is present in several circular copies of approximately 16.4kbp and it is considered rich in unmethylated CpG sequences, the target of receptors such as TLR9. mtDNA is compartmentalized within the inner membrane of the mitochondria in healthy cells. However, in some pathologies, cell death events, or mitochondrial stress, mtDNA escapes from mitochondrial boundaries and becomes available in the cytosol or extracellular environment [15,16]. An overview of the mechanisms that lead to the presence of mtDNA outside the mitochondria has been extensively recently reviewed [17].

mtDNA can activate immune responses after being extruded in classically inflammatory cell death pathways such as necrosis, pyroptosis or necroptosis. 

Necroptotic cell death can decrease pathogen replication, but it may also release damaged mitochondria in this process [18] which possibly increases the level of mtDNA and inflammation. Likewise, necroptosis is capable of induce lung pathogenesis. It perturbs the bronchial epithelial integrity, as seen in mice infected with influenza [19], and, in humans, necrotic cell death in response to H1N1 infection is related to Acute Respiratory Distress Syndrome (ARDS) [20]. Curiously, necroptosis can be induced by accessory protein open reading frame 3a (Orf3a), presented in SARS-CoV [21], an protein also present in the SARS-CoV-2 genome. Although it has been shown that SARS-CoV-2 Orf3a can induce apoptosis, it is not clear whether it can also stimulate necroptosis [22]. Besides, mtDNA has been shown to be important in several other cellular events such as inducing apoptosis [23], neutrophil extracellular traps formation (NETs) [24], renal fibrosis and chronic and acute renal diseases [25,26] and in inflammatory events in the lung and hepatocytes (Figure 1) [27-29].

**Figure 2.**
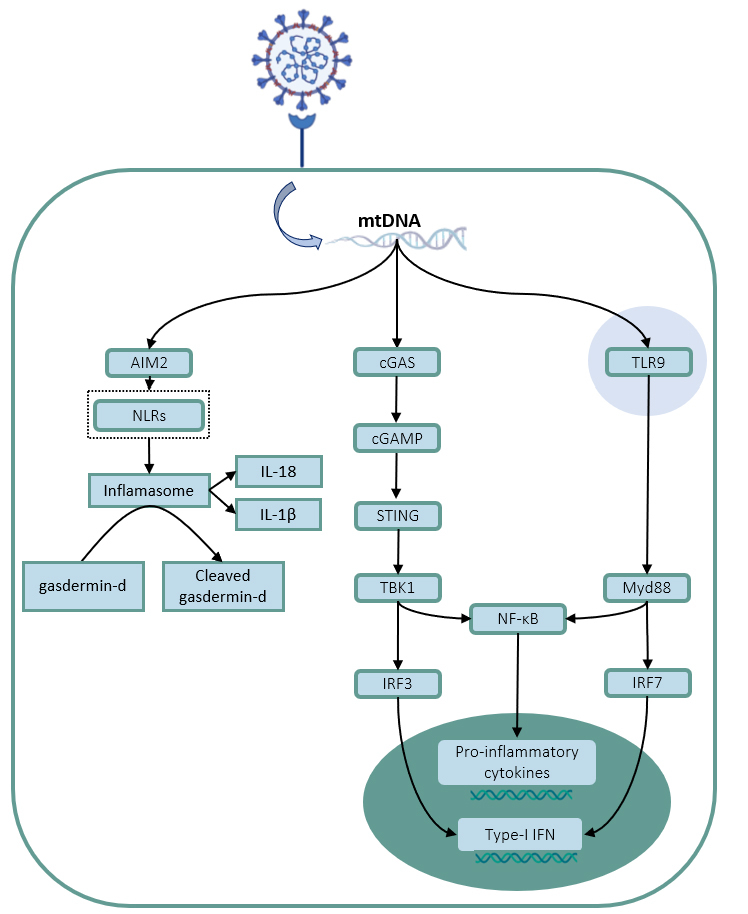
Molecular mechanisms involved in mtDNA recognition on the cytosol and endosome. SARS-CoV-2 infection leads to mtDNA releases. The TLR9, inside the endosome, recognizes mtDNA and triggers the Myd88 pathway, consequently inducing the release of pro-inflammatory factors and type-I IFNs, seen as well in cGAS pathway, but as a response of STING activation. The mtDNA is recognized by AIM2 leading to inflammasome activation, IL-18 and IL-1β production and cleave gasdermin-D.

In 2014, two distinct groups showed that, during apoptosis in a caspase 3/7 knockout model, through the permeabilization of mitochondrial membrane by pores formed by BAK and BAX, mtDNA can be released in the cytosol [30,31]. The cytosolic mtDNA activates mainly the cGAS-STING pathway (discussed below), and this leads to the induction of type I interferons (IFNs) production (Figure 2) [30,31]. It was discovered that during apoptosis, the effector caspases are responsible for suppressing the pro-inflammatory response as they can directly cleave IRF3 and cGAS, therefore inhibiting its function as a PRR, consequently decreasing the production of type I IFNs [32]. Also, it was observed that even in the presence of effector caspases, the permeabilization of the mitochondrial outer membrane by BAX-BAK gradually increases, allowing the release of mtDNA [33]. mtDNA can also be released by the action of the N-terminal portion of the gasdermin-D protein after stimulation of cells with LPS [34]. Cleaved gasdermin-D can be formed as part of the action of the inflammasome complexes [35]. Interestingly, cleaved gasdermin-D can also direct to pyroptotic cell death, thus additionally corroborating mtDNA release. This indicates that the activation of inflammasome complexes can be directly linked to the release of mtDNA in the cytosol, which may suggest a new impact of these receptors directly with the mitochondrial homeostasis, and consequently with cell metabolism.

## On mitochondria, mtDNA and SARS-CoV-2

The SARS-CoV-2 infection might be directly related with the mitochondrial status on the cells that are being infected, what could help explain why people with chronic diseases are on higher risk groups. Glycolysis and HIF-1α stabilizations have been shown to be fundamental to the replicative process of the virus in monocytes, with their inhibition abruptly decreasing the viral load in these cells [36]. Importantly, SARS-CoV-2 infected monocytes had an increased level of mitochondrial reactive oxygen species (mtROS) [36]. mtROS can directly oxidize the mitochondrial DNA, and therefore reduce mitochondrial bioenergetics and ATP supply for the cell [37]. Oxidized mtDNA and mtROS are both strong inductors of the NLRP3 inflammasome, a multiproteic complex that leads to the active forms of the cytokines IL-1β and IL-18 by cleaving pro-caspase1 into active caspase1 [38,39]. In COVID-19 patients, NLRP3 has been shown to be strictly associated with disease severity. NLRP3 is activated in patients with COVID-19 and both IL-18 and active caspase 1 can be associated with distinct levels of the disease [40]. Additionally, mtDNA when oxidized fails to undergo clearance by enzymatic repair mechanism, thus becoming more persistent in the cell and increasing their chances of activating an immune response.

SARS-CoV-2 is also proposed to have direct contact with mitochondria. SARS-CoV infection alter the functionality of the mitochondria and their accessory protein open reading frame 9b (Orf9b) is localized in the mitochondria [41]. SARS-CoV Orf3b, Orf7a and Orf8a are also in direct contact with mitochondria [42]. These proteins are mainly related to inducing apoptotic pathways in the infected cells [43,44]. Curiously, SARS-CoV-2 also presents amino acid sequences for the proteins Orf7a, Orf8a, and Orf9b, analogous to SARS-CoV [42]. In fact, a relationship of SARS-CoV-2 with mitochondria may be even more intricate, given that the predicted localization of the viral RNA is both the nucleus an the mitochondria, suggesting that this relationship is important to viral replication, with the virus being able to hijack the mitochondria and replicate inside this organelle [45]. It is very possible that if this really happens mtDNA can be easily released by exosomes, one of the main mechanism that the SARS-CoV-2 uses to exit the cell [46,47]. These circulating exosomes containing mtDNA can be a trigger to a more pronounced systemic inflammation. 

There is an association of a higher mtDNA plasma levels with ARDS in patients during critical conditions like sepsis or trauma [48]. Interestingly, circulating free mtDNA has also been shown as a predictive marker for COVID-19 pathogenesis [49]. It presented itself as a very accurate predictor of the principal severe outcomes of COVID-19, mortality, intensive care unit admission and intubation. In the article that proposed this marker, the levels of mtDNA were a more precise biomarker than the current clinically utilized, like D-dimer and C reactive Protein [49]. This data indicates that this DAMP can be, in fact, pivotal for the observed lung pathogenesis.

Regarding the interactome of SARS-CoV-2 proteins with the host cell, Gordon et al. [50] showed that the proteins Orf9c, NSP4 and NSP8 can also directly interact with mitochondria. Among the proteins found to interact with SARS-CoV-2 proteome there are mitochondrial ribosomal proteins, assembled proteins of the NADH dehydrogenase complex and proteins with intrinsic relationship with variety of mitochondrial metabolic pathways [42,50]. The article also presented results showing that a very considerable amount of the interactome was directed to pathways regarding endomembrane compartments and vesicle trafficking [50]. In addition, the SARS-CoV protein NSP3 was found to interact with the structural mitochondrial protein prohibitin (PHB) and modulate the cell survival signaling [51]. Although the result of most of the interactions presented above are still unknown, there is a clear presence of the SARS-CoV-2 in the mitochondria, inferring an important role of this organelle for the viral replication. 

## The mtDNA receptors and their role in inflammatory processes - do they constitute a possible link with SARS-CoV-2?

In viral diseases, it is thought that the activation of mtDNA receptors are not restricted only to DNA viruses, since important pathological events such as mitochondrial stress [52] and cell death in the inflammatory microenvironment are triggered with reasonable frequency in these situations. The extravasation of mtDNA into the cytosol or into the extracellular environment is observed in infections involving RNA viruses, such as dengue [53], Zika (ZIKV) [54], influenza virus [55], and even possibly in the infection caused by SARS-CoV-2 [49,56].

**Figure 3.**
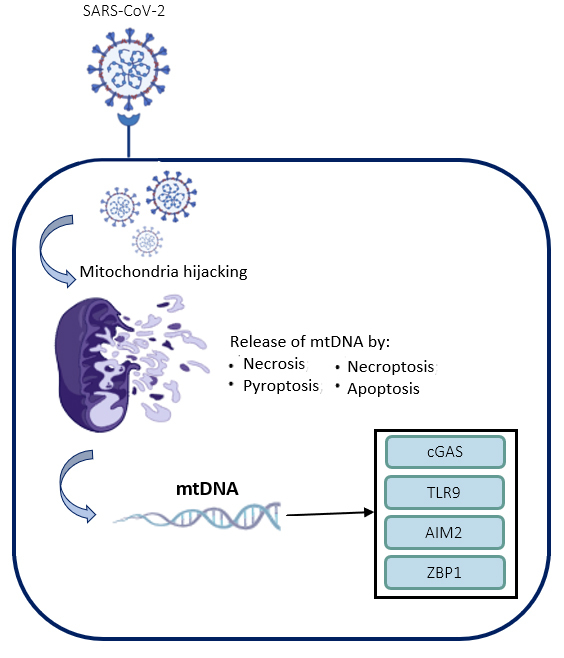
DNA receptors and SARS-CoV-2 mechanisms upon infection. Scheme showing the hijacking of mitochondria due SARS-CoV-2 entry. The mitochondria release mtDNA as consequence of different process, such as necrosis, pyroptosis, necroptosis and apoptosis. The mtDNA can be recognized by TLR9, cGAS, AIM, ZBP1.

As mtDNA presence in the cytosol can develop several different immune responses. The study of different receptors involved in mtDNA sensing can affect directly in our knowledge of the inflammatory processes. Here, we briefly describe the mechanisms involved in the receptors that already have been shown to elicit an immune response to mtDNA (Figure 3).

### Cyclic GMP-AMP synthase - stimulator of interferon genes (cGAS)

One of the main mechanisms that evolved to respond to the detection of cytosolic DNA is the protein cGAS and its downstream pathway. cGAS is currently regarded as the cytosolic DNA sensor that most corroborates with the detection and signaling of DNA - both endogenous and exogenous - as well as with the production of type I IFNs in the most diverse conditions and cell [57-60]. Upon recognizing DNA, cGAS catalyzes the formation of cyclic GMP-AMP (cGAMP) from cytosolic ATP and GTP [61]. The cGAMP produced by cGAS is a peculiar cyclic dinucleotide containing mixed phosphodiester linkages, connecting the 2’ hydroxyl and 5’ phosphate regions of GMP, with the 5 ‘phosphate and 3’ hydroxyl regions of AMP, respectively [58, 60-62]. Once recognized it leads to the activation of STING, an endoplasmic reticulum protein. After being activated STING leads to the activation of the transcription factors Interferon Responsible Factor 3 (IRF3) and NF-kB and to the induction of type I IFNs and inflammatory cytokines, respectively [60,63-65]. 

The conformational mechanisms that activate STING, as well as those that lead to the recruitment of transcription factors in its pathway, are not yet fully established. It was shown that STING needs to be phosphorylated in its Ser366 residue by the Tank Binding Kinase 1 (TBK1), and that TBK1 is also responsible for the subsequent phosphorylation and activation of the IRF3 transcription factor [57,63,66,67]. Additionally, STING can recruit IKK kinase complex and lead to phosphorylation and translocation of NF-kB to the nucleus (Figure 2) [57]. 

The extravasation of mtDNA into the extracellular environment, or into the cytosol is a process seen in different RNA viruses. Therefore several viral strategies have emerged to try to circumvent the cGAS-STING pathway [68]. The Dengue Virus for example is capable of expressing the NS2B protease, responsible for leading to lysosomal degradation of cGAS [69], ZIKV is also capable of degrading cGAS by the action of NS1 and caspase-1 [54], and SARS-CoV and HCoV-NL63, two coronaviruses, express papain-like proteases capable of inhibiting STING-mediated IRF3 activation [70]. Interestingly, the SARS-CoV-2 virus also expresses a similar papain-like protein [71]. 

Still, in a more general context, it was recently discovered that the cytokine IL-1β, present in a plethora of inflammatory processes and derived from inflammasome cleavage of pro-IL-1β, is capable of inducing the release of mtDNA and consequent activation of the cGAS-STING pathway in pulmonary epithelial cells A549 and the myeloid lineage THP-1 [72]. Additionally, Gkirtzimanaki et al. [73] also showed that INF-α leads the release of mtDNA in monocytes derived from patients with lupus. In this case, cytosolic mtDNA is derived from changes in mitochondrial metabolism, inducing an increase in ROS and lysosomal pH, culminating in an inefficient mitophagy in the monocytes analyzed. mtDNA in this case also induces the cGAS-STING pathway and leads to an increase in the cellular inflammatory profile [73]. Although in this case patients with untreated lupus were compared with healthy controls, representing in this a condition of chronic inflammation vs homeostasis, it would be important for a better understanding of the immune system to delimit the temporal variable and what is the necessary basal level of chronic inflammation for this phenotype to be observed. These responses may indicate fundamental insights into diseases where type I IFNs are important, as apparently is the case with SARS-CoV-2 disease.

### Toll-like 9 (TLR9)

TLR’s comprehend a family of several membrane associated PRRs capable of sensing a variety of different stimuli, ranging from pathogen associated molecular patterns like LPS to damage associated molecular patterns like extracellular ATP. TLR9 is normally present in endosomes and is activated in the presence of unmethylated CpG of both double and single strand DNA structures [74]. DNA rich in unmethylated CpG structures is a common characteristic of several different pathogens as well as of mtDNA, and it is present in viruses such as Epstein-Barr virus [75], Herpes Simplex viruses (HSV-1 and HSV-2)[76,77] and Cytomegalovirus (CMV) [78]. After the recognition and binding of DNA by TLR9, the receptor dimerizes and allow the binding of the adaptor protein MyD88 [74,79,80]. This complex activates a signaling cascade that stimulates the translocation of NF-κB and IRF3 to the nucleus, leading to the expression of genes responsible for the production of cytokines and chemokines, such as type I IFNs (Figure 2) [81,82]. The detection of mtDNA by the TLR9 receptor, similar to cGAS, also leads to a plethora of different responses. The recognition of mtDNA by TLR9 in the lung induces inflammation via NF-kB pathway [83,84]. The mtDNA/TLR9 axis was also shown to be able to be important in the induction of cardiomyopathy [85], promote ischemia-reperfusion injury [86], induce apoptosis [87], muscle inflammation [88], and vascular disfunction [89,90].

### Absent in melanoma 2 (AIM2)

The AIM2 sensor was initially described in 2008 [91] after observations that macrophages deficient in the ASC protein, an essential protein for the function and activity of the inflammasome complexes, failed to induce the cytokine IL-1β and cell death after the cells were transfected with double stranded DNA (dsDNA) [91,92]. It was observed that this event did not happen to macrophages lacking the NLRP3, NLRP6 or NLRP12 inflammasome proteins, therefore giving rise to the AIM2 function [92]. 

AIM2 is a multimeric cytoplasmic sensor, belonging to the AIM2 like receptors protein family [93] and is able to recognize double-stranded DNA both from self, viruses and bacteria, and thus form the inflammasome complex. AIM2 is able to induce the active forms of caspase-1 and the cytokines IL-1β and IL-18 and also lead to cell death by pyroptosis via cleavage of the gasdermin-D protein [94]. 

AIM2 can be activated independent of the DNA sequence or GC content [95,96]. Interestingly, AIM2 binds directly to the two DNA strands, both between the larger and the smaller grooves, which explains, in theory, why their activation does not occur with single-stranded DNA [96]. Interestingly, in patients with type 2 diabetes the level of circulating mtDNA is significantly higher compared to healthy patients [97]. It was observed that this extracellular mtDNA is the driver for the activation of macrophages, and the subsequent induction of the cytokine IL-1β, contributing to the establishment of chronic inflammation [97]. AIM2 has also been observed to sense mtDNA from events of mitochondrial stress, such as increased cell levels of cholesterol, which lead to the extravasation of mtDNA [98], and in non-alcoholic fatty liver disease, driving inflammation and hepatocyte pyroptosis [99].

### ZBP1

Z-DNA binding protein 1 (ZBP1), also referred as DAI (DNA-dependent activator of IFN-regulatory factors), is a cytosolic DNA sensor related to the initiation of the innate immune response. It activation can lead to programmed cell death and inflammation [100,101]. ZBP1 when associated with DNA recruits TBK1 that regulates the activation of IRF3 and induces the expression of type I IFNs [101]. ZBP1 needs oligomerization/multimerization to initiate its signaling transduction [102]. 

Murine L929 fibroblasts lacking ZBP1 express less NF-kB after infection with HSV-1, which reveals a possible importance of this sensor for activating a proinflammatory response [101]. However, the role of ZBP1 as an important DNA sensor has been questioned, as mice lacking ZBP1 (Zbp1 -/-) were able to generate an innate and adaptive immune response after DNA vaccination and after exposition to double-stranded B-form of DNA [103,104].

Recently, the ZBP1 DNA sensor was included in the select group of direct mtDNA receptors. Low levels of chronic oxidative stress in smoking-derived tissue damage models, have also been shown to be able to not only cause damage to mtDNA but also lead to its presence in the cytosol [52]. In this case, ZBP1 is capable of binding to mtDNA in pulmonary epithelial cells and induce inflammation via TBK1 and by stabilizing the IRF3 transcription factor [52].

It is of paramount importance for immunology to unveil how and when each receptor is preferentially activated to seek possible new therapeutic approaches, as well as to unravel the processes of why and how mtDNA is released from cells.

Finally, recent data is linking each one of the mtDNA receptors presented above with the SARS-CoV-2 infection.

In array analysis, the level of TLR9 was upregulated in response to SARS-CoV infection, even more than other TLRs such as TLR2 and TLR4 [105]. TLR9 can in fact explain a huge variety of the symptoms observed in COVID-19, and in specific cases be hypothesized as the leading cause of the hyperinflammatory process [106]. AIM2 can be activated in monocytes infected with SARS-CoV-2 in an antibody mediated manner, causing pyroptotic cell death [107] and in pangolins, carriers of coronaviruses and together with bats, focus of studies of the SARS-CoV-2 zoonotic transmission to humans, lack the ZBP1 protein [108]. The authors of this study hypothesize that this difference in the innate immune system is probably a selection factor for pangolins to carry out coronaviruses, switching from a mechanism of immune virus combat and resistance, to a more tolerogenic state.

Regarding cGAS, SARS-CoV-2 proteins Orf3a and 3CL are able to interact and inhibit cGAS-STING activity. Orf3a bind to STING independently of it C or N terminal regions, and inhibit NF-kB activity and downstream gene expression [109]. 3CL protein inhibit K63-ubiquitin-mediated modification of STING and also decreases the function of NF-kB. Interestingly in the study that reported this results, both Orf3a and 3CL did not interfered with IRF3 activity [109].

## Conclusion

The relationship of mitochondria with the immune system is complex as different mitochondrial dynamics and metabolic pathways result in very different phenotypes of immune cells [7]. In addition, mitochondria can serve as inner DAMP generator, releasing ATP, mtDNA, and other molecules that can activate the immune system [10]. We have focused in the present study in the mtDNA, which have been shown to elicit a powerful proinflammatory response upon its detection. A large number of processes can be responsible for its extrusion of the mitochondrial compartment, including a variety of RNA viruses that have even evolved mechanisms to circumvent the mtDNA detection. 

As novel functions and interactions are discovered almost on a daily basis, the receptors here reviewed can possibly contribute to the understanding of the inflammatory process in the SARS-CoV-2 lung pathogenesis. The presence of mtDNA in cytosol or in the extracellular environment is highly associated with an exacerbated inflammation in the lungs, one of the principal clinical manifestations of SARS-CoV-2 [28,110-113]. Furthermore, a vast number of the receptors reviewed here are directly associated with lung inflammatory diseases [111,112]. Additionally, each of the receptors reviewed here have presented significant evidence of relationship with COVID-19. The release and presence of mtDNA outside mitochondria due to SARS-CoV-2 infection might reflect one important pathogenic mechanism and exploring the role of mtDNA in clinical patients together with its receptors can be beneficial to unravel a new mechanism of the disease and to open new treatment possibilities.

### Abbreviations

ACE2: angiotensin-converting enzyme 2; AIM2: absent in melanoma 2; AMP: adenosine monophosphate; ARDS: acute respiratory distress syndrome; ASC: caspase recruitment domain; ATP: adenosine triphosphate; BAK: pro-apoptotic Bcl-2 family member; BAX: pro-apoptotic Bcl-2 family member; cGAMP: cyclic GMP-AMP; cGAS: cyclic GMP-AMP (cGAMP) synthase; CgP: 5'-C-phosphate-G-3’; CMV: cytomegalovirus; COVID-19: corona virus disease 19; CpG: cytosine-phosphate-guanine; DAI: DNA-dependent activator of IFN-regulatory factors; DAMP: damage-associated molecular pattern molecule; DNA: deoxyribonucleic acid; dsDNA: double-stranded DNA; GC: guanine-cytosine; GMP: guanosine monophosphate; GTP: guanosine triphosphate; H1N1: human influenza A strain; HCoV-NL63: Human coronavirus NL63; HIF-1α: hypoxia-inducible factor-1 alpha; HSV-1: herpes simplex virus type 1; HSV-2: herpes simplex virus type 2; IFN: interferon; IFN-α: interferon alpha; IKK: inhibitor of nuclear factor‐κB (IκB) kinase; IL-18: interleukin 18; IL-1β: interleukin 1 beta; IRF3: interferon responsible factor 3; LPS: lipopolysaccharides; MAVS: mitochondrial antiviral-signaling protein; mtDNA: mitochondrial DNA; mtROS: mitochondrial reactive oxygen species; MyD88: myeloid differentiation factor 88; NADH: nicotinamide adenine dinucleotide reduced; NET: neutrophil extracellular traps; NF-kB: nuclear factor kappa B; NLRP12: NOD-like receptor family pyrin domain containing 12; NLRP3: NOD-like receptor family pyrin domain containing 3; NLRP6: NOD-like receptor family pyrin domain containing 6; NS1: nonstructural protein 1; NS2B: nonstructural protein 2B; NSP3: nonstructural protein 3; NSP4: nonstructural protein 4; NSP8: nonstructural protein 8; Orf3a: open reading frame 3a; Orf7b: open reading frame 7b; Orf8b: open reading frame 8b; Orf9b: open reading frame 9b; Orf9c: open reading frame 9c**;** PAMP: pathogen associated molecular pattern; pH: potential hydrogen; PHB: prohibitin; PRR: pattern recognition receptors; RIG-I: retinoic acid-inducible gene I; RNA: ribonucleic acid; ROS: reactive oxygen species; SARS-CoV: severe acute respiratory syndrome coronavirus; SARS-CoV-2: severe acute respiratory syndrome coronavirus 2; Ser366: serine residue 366; STING: stimulator of interferon genes; TBK1: TANK-binding kinase 1; TLR: toll-like receptor; TLR2: toll-like receptor 2; TLR4: toll-like receptor 4; TLR9: toll-like receptor 9; TMPRSS2: transmembrane serine protease 2; ZBP1: Z-DNA binding protein 1; Zikv: Zika virus.
